# The effects of senior brain health exercise program on basic physical fitness, cognitive function and BDNF of elderly women - a feasibility study

**DOI:** 10.20463/jenb.2016.06.20.2.2

**Published:** 2016-06-30

**Authors:** Jung-Eun Byun, Eun-Bum Kang

**Affiliations:** 1 Sports·Wellness Research Center, Yongin University, Yongin Republic of Korea; 2 Exercise Biochemistry Laboratory, Korea National Sport University, Seoul Republic of Korea

**Keywords:** Senior brain health exercise program, Basic physical fitness, Cognitive function, Brain derived neurotrophic factor

## Abstract

**[Purpose]:**

This study was to investigate the impacts of senior brain heath exercise (SBHE) program for 12 weeks to basic active physical fitness, cognitive function and brain derived neurotrophic factor (BDNF) in elderly women.

**[Methods]:**

Subject of this study is total of 24 women in the age of 65-79 who can conduct normal daily activity and communication but have not participated in regular exercise in recent 6 months. The study groups were divided into an exercise group (EG, n=13) and a control group (CG, n=11). The exercise program was consisted of SBHE, and training frequency was 4 times weekly, of which training time was a total of 50 minutes each time in level of intensity of 9-14 by rating of perceived exertion (RPE).

**[Results]:**

First, 12-week SBHE program has shown statistical increase in basic physical fitness in the EG comparing with the CG, such as lower body strength, upper body strength and aerobic endurance, but not in flexibility, agility and dynamic balance. Second, in the case of Mini-mental state examination Korean version (MMSE-K) and BDNF, it showed that there was a statistically significant increase in the EG comparing with the CG.

**[Conclusion]:**

In this study, 12-week SBHE program has resulted in positive effect on change of basic physical fitness (strength and aerobic endurance), cognitive function and BDNF. If above program adds movements that can enhance flexibility, dynamic balance and agility, this can be practical exercise program to help seniors maintain overall healthy lifestyle.

## INTRODUCTION

Aging is the process emerged by accumulated harmful changes in terms of physical and physiological aspects during entire life^1,2^. As getting old, there comes diverse and distinctive, cognitive changes. Among them, environmental factors and lifestyle become to influence process of aging alongside with functional changes due to biological effect^3^. The elderlies become to experience decrease in amount of physical and social activities due to these physical, physiological and cognitive changes. The female elderlies in particular show higher frequency of chronic diseases with decrease in mobility and daily activities compared to male elderlies^4^, which requires improvement of life style in order to solve health problems in female elderlies. The aging effect in particular is related to dementia with cognitive disorder^5^, Recently prevalence of which in South Korea has consistently increased and been reported as higher in women compared to men^6^.

Generally decrease in physical strength such as muscular force, flexibility, agility balance, etc., begins to emerge as getting old and leads to falling, fatigue and decreased motor skill, which prevent elderlies from health and independent daily life^7,8^. Decrease in cardio pulmonary function in particular was reported to damage cerebral cortex and causes central nervous system disorders to consequently decrease overall function of brain^9,10^, implying physical strength as an important factor in maintenance of brain function. In short, as physical strength is involved with daily life performance of elderlies and plays important role in onset of degenerative nervous disorder, dementia, bringing need of exercise to the fore as a method to solve health issues in elderlies associated with physical health and brain.

The human brain responds to a lot of environmental factors and various experiences to show structural and functional changes called neural plasticity^11^. However, such coping skill of the brain decreases with aging; this aging process causes decline of brain function. In other word, overall cognitive capability including attention, memory, working memory, communication ability, etc. decreases by decreasing neural plasticity. Moraes *et al*^12^ reports that exercise increases blood flow rate of the brain as well as supply of oxygen to the brain, resulting in increase in neural plasticity at cerebellum and hippocampus. It means that increase in synapsis of nerve cells more easily induces neural plasticity based on factors like environment, learning and experience of rich stimulus^13, 14^. In relation to neural plasticity, the brain-derived neurotrophic factors (BDNFs) are known to express at the hippocampal site, influence survival and growth of nerve cells and have close relationship with cognitive functions including learning, memory, etc.^15^. In addition, expression of BDNF is known to decrease by 70% in patients with Alzheimer’s disease^16^. Referring to previous studies reporting potential increase in express of BDNF by exercise, the studies to confirm relationship between expression of BDNF by exercise and cognitive function are ongoing^17,18^. However, a number of studies reporting no effect of exercise on expression of BDNF raises need to conduct further study accordingly.

While variety of approaches are currently attempted to prevent dementia worldwide, exercise is now considered as one of the most definite and safe way for anti-aging and prevention of dementia^20^. According to meta-analysis of exercise-related literatures, the aerobic exercise is reported to have positive effect on maintenance of elderlies’ cognitive functions and be more effective to delay decline of cognitive function if adding muscles and flexibility exercises in particular^21^. Recently as the way to improve elderlies’ health issues in South Korea, studies using various types of exercise programs such as elastic band, aerobic and complex exercises are currently on going^7,16,22,23^. These studies demonstrating effect of exercise program to prevent decline of daily life activities and brain function in elderlies suggest that regular and consistent exercise in long-term basis develops brain structure and cognitive function and prevents brain disorders^24^, and that the improved physical strength has positive effect on brain structure as well as function.

Although a number of studies demonstrating positive role of exercise in brain health of elderlies have been reported, most of exercise programs with the experimentally verified effects consist of repeated simple motion, walk and equipment exercise (elastic band) which do not reflecting consideration of physical characteristics or cognitive capacity specific to elderlies. In addition, it is suggested as the low intense level of program for elderlies in terms of exercise intensity, time and frequency generally applied, but lack diversity of applicability in terms of exercise type. It also is difficult to expect elderlies’ consistent participation in and use of such exercise program due to lack of interest and expectation of elderlies and their instructors. Therefore, there needs to develop exercise program reflecting physical characteristics, physical strength and cognitive function factors of elderlies and available as various methods and attempt to put an effort to induce continuous participation of elderlies in multilateral directions.

Accordingly, in this study, the Senior Brain Health Exercise (SBHE) program for elderlies was developed as an effort to supply helpful program for improvement of physical strength and cognitive function necessary for daily activities of elderlies. The SBHE program consists of pre-organized motions considering physical and cognitive factors of elderlies; it is music-based exercise with regular and repetitive patterns, which creates consecutive motion of exercise following the specific music according to the type of exercise in GX program^25,26^. The exercise participant expresses physical motions according to language, hand signal and direction of the instructor, while the exercise instructor proceeds with the exercise class according to the procedure of exercise performance to reach the pre-defined final target, inducing the participant to achieve the exercise target via cognition, learning and automation (training) stages^27^. These characteristics of SBHE program are expected to help improvement of cognitive functional as well as physical strength of elderlies, by repeated remembering and combining motions.

Therefore, objective of this study, as a pilot study for large-scale experimental study, was to develop and supply the SBHE program for improvement of physical strength and cognitive function necessary for daily activity performance by elderlies. For this purpose, effect of SBHE program on change of physical fitness, cognitive function and BDNF in female elderlies was examined.

## METHODS

### Subjects

In this study, study subjects were selected from two community welfare centers located at S city, which permit study participation. Among 65-79 years old elderlies with capacity for daily activities including communication, self mobility, etc. the elderlies who had no regular exercise experience in recent 6 months, was able to do exercise and was willing to actively participate in study exercise for this study were selected. The final 30 subjects were determined, who provided signed consent form after hearing explanation about objectives and methods of the study, confidentiality of study data and voluntary withdrawal of participation. The experiment was conducted in the determined subjects who were divided into the exercise group (n=15) and the control group (n=15). except 6 subjects who did not attend the exercise program more than 5 times of total 51 times, who did not attend to secondary measurement, who withdrew from the study for personal reasons such as hospitalization and moving, etc., data obtained from total 24 subjects in the exercise group (n=13) and the control group (n=11) were used in analysis. Physical characteristics of subjects are provided in <Table 1>.

**Table 1.** Physical characteristics of subjects

Group	Age(yrs)	Height(cm)	Weight(kg)	BMI(kg/m2)
CG(n=11)	70.46±2.85	155.2±3.19	59.89±3.55	24.86±1.19
EG(n=13)	70.45±4.18	153.56±5.15	56.93±5.53	24.11±1.71

Values are Mean and SD. CG: Control Group; EG: Exercise Group

### Senior brain health exercise program

This exercise program consisted of stages to recognize and perform single motion and thereafter to achieve complex motion combining several single motions. In addition, it was designed with consultation by professors specialized in geriatric physical education and experts in geriatric healthcare exercise, and modified and supplemented according to overall characteristics of the subject based on his/her heart rate and exercise awareness measured by preliminary experiment in order to be applied to this experiment. The SBHE program was intend for the subject to sequentially achieve exercise task at cognition, combination and automation (training) stage suggested in 3-stage exercise performance model by Fitts & Posner^27^ and the instructor directed each exercise motion according to the procedures for language and behavior instruction, task demonstration and task instruction.

**Figure 1.**
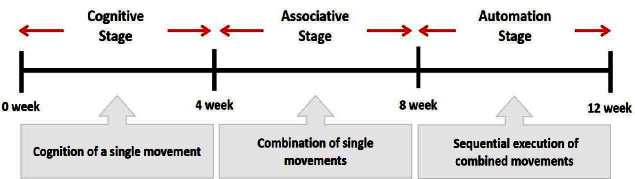
Three stage of exercise performance

The subjects in the exercise group understood and acquainted the pre-organized motions and reached to the automation stage where the Group Exercise (GX) that instructor led the subjects to follow the pre-organized routine with repetitive and regular pattern was applied. The GX program was the process gradually complexing motions of upper and lower limbs from basic (cognition) to applicable (combination) level by using music to create and remind change in movement , which finally induced active and continuous participation in the program based on interest arose from the participants. Details of 3-stage exercise performance are provided in <Fig. 1>.

Intensity of appropriate exercise was set to prevent injuries and maximize effect of exercise, which played an important role in physical fitness as well as cognitive ability. Moderate intensity of aerobic exercise has positive effect on improvement of physical strength for daily activities as well as cognitive function in elderlies^28^, and has been reported as more effective for improvement of cognitive function and working memory by increasing BDNF in variety of age groups^29^. Accordingly, the SBHE program was implemented 50 minutes daily,4 times (Monday, Tuesday, Thursday, Friday) weekly for 12 weeks with 9-14 RPE of moderate-high intensity.

The contents of this program consisted of communiThe contents of this program consisted of communication, warm-up, main exercise and warm-down in order, based on physical strength factors such as aerobic exercise beginning with walking not to cause excessive orthopedic stress, muscle strengthening exercise, balancing exercise involved with falling and mobility issues^30^ and agility balance exercise required for sudden quick action^31^ and cognitive function factors such as linguistic capacity, orientation, comprehension, judgement, attention, etc. 13 motions in this exercise was repeated as a single motion in both directions at the cognition stage, and the number of repeating single motion was gradually reduced by adding the combined 2-3 specific motions while increasing the number of repeating combined motion at the combination stage. At the training stage, the speed of motion gradually increased from slow to fast motion, based on the pattern of motion at the combined stage. Various modified motions were performed by moving and switching to each direction of the body. Details of structure and pattern of motion of SBHE program are provided in <Table. 2> and <Table. 3>, respectively.

**Table 2.** Configuration of Senior Brain Health Exercise Program

Categories	Contents	Time(50min)	RPE	HR(%)
Communication	Situation assessment	5		
	Responding to language instruction			
	Counting with hands			
	Drawing with hands			
	Following hand signal			
Warm-up (Relaxation)	Head rotating	5	7~8	50~60
	Arm movement			
	Leg movement			
	Torso movement			
	Calisthenics			
Main exercise (Strengthen)	Walking	30	9~14	60~80
	Stepping			
	Knee up			
	Leg up			
	Hopping			
	Jumping jack			
	Collecting breath			
	Squatting			
	Lift the heels			
	Turning the leg			
	Raising the arm			
	Bending the wrist			
	Balancing			
Cool-down (Relaxation & Recovery)	Calisthenics	10	7~8	50~60
	Stretching			

**Table 3.** Movements Pattern of Senior Brain Health Exercise Program

Contents	Movements			
I. Communication				
1. Situation assessment	① Assessing recognition of self ② Assessing recognition of time ③ Assessing recognition of place			
2. Responding to language instruction	① Answering name of objects ② Articulating words ③ Speaking words backwards			
3. Counting with hands	① Counting 1 to 10 starting with right thumb ② Counting 10 to 1 starting with left little finger ③ Counting per teachers instruction	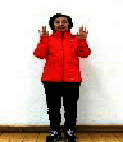	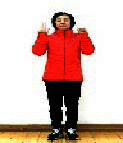	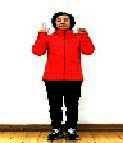
4. Drawing with hands	① Drawing figures ② Drawing objects ③ Drawing as instructed	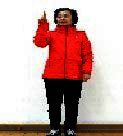	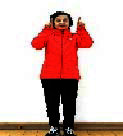	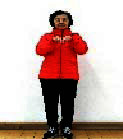
5. Following hand signal	① Following hand count ② Following hand direction ③ Following hand movement	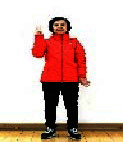	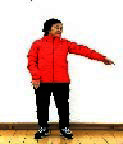	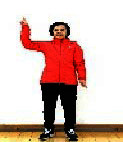
II. Warm-up (Relaxation)				
1. Head rotating	① Looking at different directions ② Rotating the head ③ Looking at directions as instructed	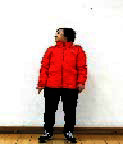	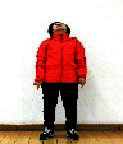	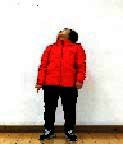
2. Arm movement	① Bending and straightening each or both arms ② Rotating each or both arms	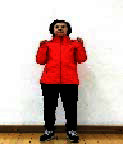	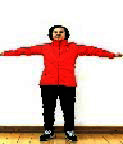	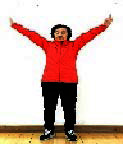
3. Leg movement	① Bending and straightening a leg ② Rotating a leg	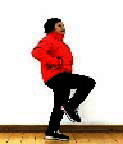	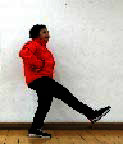	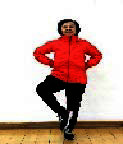
4. Torso movement	① Roll down and roll up ② Forward bend with arms stretched out ③ Rotating torso	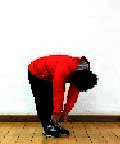	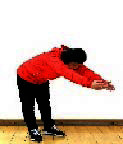	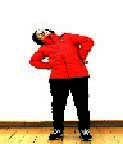
5. Calisthenics	① Drawing curves with tissue ② Passing a tissue from one hand to the other hand ③ Throwing and catching a tissue	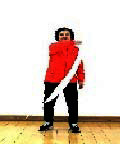	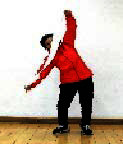	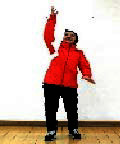
III. Main exercise (Strengthen)				
1. Walking Head rotating	① Walking in place and around ② Cross walking ③ Walking in circle	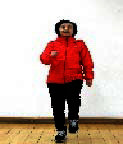	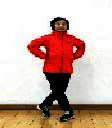	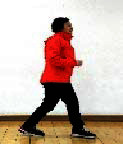
2. Stepping	① Stepping to the side ② Stepping in different direction	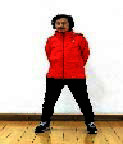	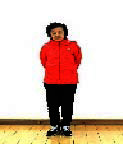	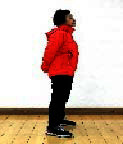
3. Knee up	① Knee up ② Knee up in different direction	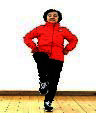	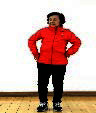	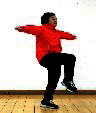
4. Leg up	① Raising leg with straight knee ② Raising leg with straight knee in different direction	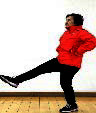	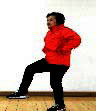	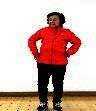
5. Hopping	① Hopping ② Hopping in different direction	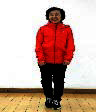	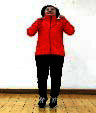	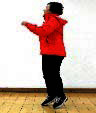
6. Jumping jack	① Jumping Jack to front and back ② Jumping Jack to side	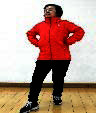	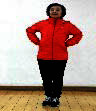	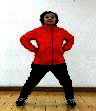
7. Collecting breath	① Collection breath while walking ② Collecting breath using hands	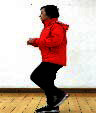	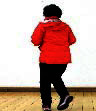	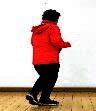
8. Squatting	① Squatting in slow and fast pace ② Step squat ③ Squat hold for 4 count	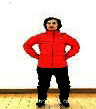	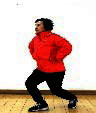	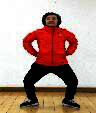
9. Lift the heels	① Lift the heels while feet apart ② Lift the heels while feet together ③ Squatting variation with heels up	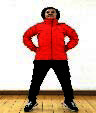	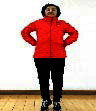	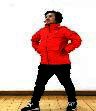
10. Turning the leg	① Rotating the leg with bent knee ② Rotating the leg with straight knee	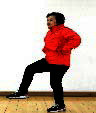	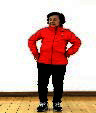	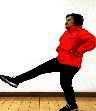
11. Raising the arm	① Raising an arm to shoulder height ② Raising the both arms to shoulder height	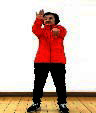	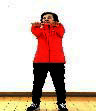	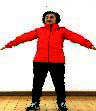
12. Bending the wrist	① Bending the wrist with raised arms ② Turning the wrist with raised arms ③ Twisting with raised arms ④ Rotating the raised arms	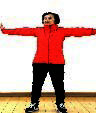	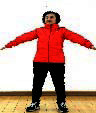	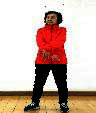
13. Balancing	① Balancing with raised leg ② Balancing on one leg with the other foot on standing leg ③ Hands in prayer position, on the side and over the head	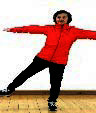	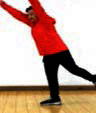	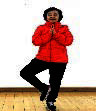
IV. Cool-down (Relaxation & Recovery)				
1. Calisthenics	① Rotating arms, legs and torso	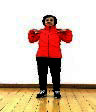	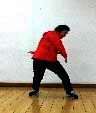	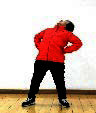
2. Stretching	① Stretching ② Relaxing body and mind	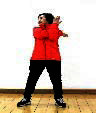	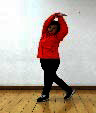	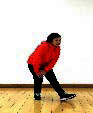

### Basic physical fitness

Physical fitness was measured by geriatric physical strength test developed by Rikli & Jones^32^. The geriatric physical strength test is a tool to measure 6 domains including muscular endurance (upper and lower body), aerobic endurance, flexibility (upper and lower body), dynamic balance and agility, based on the factors required for independent life and active physical activities of elderlies. The subjects performed preliminary exercise before measurement, and were well informed of precautions through prior practice of each exercise item. The assistant was accompanied by subjects in order to prevent negligent accident. The subjects were instructed to perform warm-down exercise such as self-stretching and walking upon completion of each item measurement.

Firstly, muscle endurance of lower body was measured by sitting at-and-standing from the chair; the maximum number of sitting-and-standing was measured while both arms were being crossed on the chest and the back being stretched. Muscle endurance of upper body was measured by dumbbell raising; the subjects were instructed to hold 2 kg dumbbell by one hand with stronger strength while sitting on the chair, stretching the upper body and making a long arm, and then to bend the elbow to rotate the wrist to make the palm looking the body and this motion was repeated. Muscle endurance was measured once for 30 seconds and the maximum number of motion was recorded.

Flexibility of lower body was measured by lowering upper body; the subjects were instructed to bend the knee of one leg while stretching the knee of other leg forward as much as possible. Then, the foot of stretched leg was bended by approx. 90° towards the body with the heel stepping on the ground and the upper body was slowly lowered with folded hands stretching to the point of foot. Better result was recorded; the distance from the point of middle finger to the surface of shoe was measured, while maintaining the posture for 2 seconds. In order to measure flexibility of upper body, the subjects were instructed to stand in place while straightening upper body, bend more flexible arm towards the back of the shoulder to make the palm facing down and raise the opposite arm from the waist to the upper back while making the palm facing up. Among arms, the target arm for measurement is that the middle finger of hand can more easily reach to the middle of back while straightening fingers. Flexibility was measured twice in up to 0.5 cm unit.

Aerobic endurance was measured by 2-minite marking time test; the subjects were instructed to repeat the motion raising the knees higher than between iliac crest and patella at standstill position. The number of motion completed for 2 minutes was determined once.

Dynamic balance and agility was measured by standing up from the chair, reaching to and turning around the cone and coming back to the chair. The subjects were instructed to sit on the chair while straightening the back and put both hands on the thighs. One foot was put in the front than other foot while leaning the body forward. Upon start of measurement, the subjects stood up from the chair, reached to the cone placed at 2.44 m distance, turned around the cone, came back to and sit on the chair. Alertness and dynamic balancing were measured twice and the results were recorded up to 0.1-second unit.

### Measurement of cognitive function

The cognitive test was performed by using the Mini-Mental State Examination-Korean version (MMSE-K) questionnaire modified and supplemented by Kwon & Park^34^ while reflecting Korean circumstances, from the measurement tool developed by Folstein & McHugh^33^. The MMSE-K questionnaire consists of 11 items in total 5 domains: 10 points scale of orientation (time and place), 6 points of memory (registration and recall), 5 points of attention/count ability (numeracy), 7 points of language ability (remembering names, 3-stage order, copy and repetition) and 2 points of comprehension/judgement. 1 and 0 point is obtained for each correct and incorrect answer respectively and the sum of each point is used in assessment. The highest point is 30 and the scare of point ranges: >24 = ‘normal; 20-23 = ‘dementia suspected’; 15-19 = ‘mild dementia suspected’; and <14 = ‘severe dementia suspected’. The investigator visited Dementia Supporting Center located in G-gu, Seoul to conduct measurements for this study with assistance from the professional researcher after being well informed of method and precaution of measurement. Measurement was proceeding with 1:1 interview, which the investigator made verbal inquiries, and then the subject gave an verbal answer or was assessed for his/her behavior by the investigator.

### Measurement of BDNF

10 mL blood was collected from the brachial veins of all subjects in 10-hour fasting condition, transferred into the tube, placed at room temperature for about 1 hour and centrifuged (Beckman, U.S.A, 3000rpm) for 10 minutes. The resulting supernatant, serum was transferred into the other tube and stored at –80°C for the next analysis. Serum BDNF analysis was conducted by using R&D system (mineapolis, MN, U.S.A) made by Human BDNF Quantikine ELISA Kit (Catalog DBD00) and according to the experimental method recommended by the manufacturer.

### Statistical analysis

Descriptive statistics (mean±SD) was calculated from the collected data by using SPSS 18.0 program and twoway repeated measures ANOVA was conducted to verify difference in physical fitness (muscle strength of lower and upper limbs, flexibility of lower and upper extremities, aerobic endurance and agility balance), cognitive function (MMSE-K) and brain-derived neurotrophic factor (BDNF) between groups (exercise group and control group) and between periods (before and after exercise). In order to specifically confirm interacting effect, comparison was carried out based on independent t-test per group and paired t-test per period. Significance level was set as α=.05.

## RESULTS

### Change in basic physical fitness

The results of descriptive statistic as well as analysis of variance for physical fitness are provided in <Table 4>.

**Table 4.** Change of basic physical fitness

Variable	CG		EG		F	p
	Pre	Post	Pre	Post		
Lower-limb muscle endurance (times/30 sec)	15.10±1.51	14.73±1.56	14.39±1.76	15.31±1.03	a: .012 b: 1.494 c: 7.901	.912 .235 .010*
Upper-limb muscle endurance (times/30 sec)	19.55±2.38	19.00±2.53	18.62±2.06	21.00±2.45	a: .359 b: 6.352 c: 16.122	.555 .019* .001**
Lower-body flexibility (cm)	10.83±2.78	11.35±2.47	9.49±2.76	10.27±2.55	a: 1.259 b: 23.342 c: .932	.274 .000*** .345
Upper-body flexibility (cm)	-6.41±4.83	-6.06±4.86	-5.05±5.54	-4.48±5.62	a: .468 b: 10.553 c: .599	.501 .004** .447
Aerobic endurance capacity (times/120 sec)	88.45±6.79	87.64±7.07	90.08±5.57	91.69±4.53	a: 1.386 b: .739 c: 6.887	.252 .339 .015*
Agility·Balance (sec)	5.48±0.52	5.49±0.60	5.72±0.51	5.60±0.44	a: .718 b: 1.714 c: 2.463	.406 .204 .131

Values are means and SD. CG: Control Group; EG: Exercise Group

a: group, b: time, c: group*time

*P<.05; **P<.01; ***P<.001 *significant main effect and/or interaction

As a result of analysis of variation for mean and standard deviation of lower-limb muscle endurance, there was difference in interacting effect [F_(1,22)_=7.901] at p=.01. There was no statistically significant difference in the control group, while there was statistically significant increase compared to pre- test in the exercise group, as a result of paired t-test (t=-2.803, df=12, p=.016).

As a result of analysis of variation for mean and standard deviation of upper-limb muscle endurance, there was difference in interacting effect [F_(1,22)_=16.122] at p=.001. There was no statistically significant difference in the control group, while there was statistically significant increase compared to pre- test in the exercise group, as a result of paired t-test (t=-5.029, df=12, p=.001).

As a result of analysis of variation for mean and standard deviation of lower-body flexibility, there was no difference in interacting effect [F_(1,22)_=0.932] at p=.345. Therefore, there is difference in interacting effect [F_(1,22)_=23.342] between periods at p=.001 while there was no difference in interacting effect [F_(1,22)_=1.259] between groups at p=.274, as a result of test on main effect.

As a result of analysis of variation for mean and standard deviation of upper-limb flexibility, there was no difference in interacting effect [F_(1,22)_=0.599] at p=.447. Therefore, there is difference in interacting effect [F_(1,22)_=10.553] between periods at p=.004 while there was no difference in interacting effect [F_(1,22)_=0.468] between groups at p=.501, as a result of test on main effect.

As a result of analysis of variation for mean and standard deviation of aerobic endurance capacity, there was difference in interacting effect [F_(1,22)_=6.887] at p=.015. There was no statistically significant difference in the control group, while there was statistically significant increase compared to pre- test in the exercise group, as a result of paired t-test (t=-2.880, df=12, p=.014).

As a result of analysis of variation for mean and standard deviation of dynamic balance and agility, there was no difference in interacting effect [F_(1,22)_=2.463] at p=.131. Therefore, there is no difference in interacting effect [F_(1,22)_=1.714] between periods at p=.204 while there was no difference in interacting effect [F_(1,22)_=0.718] between groups at p=.406, as a result of test on main effect.

### Change in MMSE-K score

The results of descriptive statistic as well as analysis of variance for change in MMSE-K point by 12-week SBHE program are provided in <Table 5>. As a result of analysis of variation for mean and standard deviation of MMSE-K point, there was difference in interacting effect (F_(1,22)_=5.587) at p=.027. There was no statistically significant difference in the control group, while there was statistically significant increase compared to pre- test in the exercise group, as a result of paired t-test (t=-2.856, df=12, p=.014).

**Table 5.** Change of MMSE-K score

Variable	CG		EG		F	p
	Pre	Post	Pre	Post		
MMSE-K (score)	25.19±1.83	25.09±2.12	26.23±1.64	27.08±2.10	a: 3.934 b: 3.629 c: 22.000	.060 .070 .027*

Values are means and SD. CG: Control Group; EG: Exercise Group

a: group, b: time, c: group*time

*P<.05; **P<.01; ***P<.001 *significant main effect and/or interaction

### Change in BDNF concentration

The results of descriptive statistic as well as analysis of variance for change in BDNF concentration by 12-week SBHE program are provided in <Table 6>. As a result of analysis of variation for mean and standard deviation of BDNF concentration, there was difference in interacting effect (F_(1,22)_=7.688) at p=.011. There was no statistically significant difference in the control group, while there was statistically significant increase compared to pre- test in the exercise group, as a result of paired t-test (t=-3.007, df=12, p=.011).

**Table 6.** Change of BDNF Concentration

Variable	CG		EG		F	p
	Pre	Post	Pre	Post		
BDNF (ng/mL)	18.99±1.69	18.80±2.34	19.07±1.51	20.14±1.31	a: 1.123 b: 3.633 c: 73688	.301 .070 .011*

Values are means and SD. CG: Control Group; EG: Exercise Group

a: group, b: time, c: group*time

*P<.05; **P<.01; ***P<.001 *significant main effect and/or interaction

## DISCUSSION

Ultimate purpose of SBHE program development done in this study was improvement of cognitive function based on improvement of effective physical fitness. To achieve this purpose, the SBHE program was designed based on the contents of geriatric cognitive function measure, MMSE-K test, and geriatric physical strength factors. The exercise instructor demonstrated instructive language and behavior and gave a direction to the participant following the GE method^25^, while the participant recognized and combined the motions according to the directions of instructor, reached to the automation stage^27^ to repeat those motions and finally achieved the target exercise. Modifications and supplements are required as the following, based on the effect of 12-week SBHE program on basic physical fitness, cognitive function and brain derived neurotrophic.

First of all, when looking at the effect of SBHE program on physical fitness, lower- and upper limb muscle endurance and aerobic endurance capacity (except flexibility and agility balance) were improved in the exercise group, compared to the control group. The SBHE program applied to this study was modified to set the intensity of exercise based on measurements of RPE and heart rate for safe and effective exercise learning and customized exercise for each participant and to diversify intensity of exercise motions. The pattern of motions was organized to start with simple, basic motions and gradually proceed with complex motions by revising one factor at a time, so it could improve muscle balance by using various muscle sets. In addition, motions were combined by switching directions and induced to use variety of muscular contractions by switching from rapid motions to slow and moderate motions. The instructor appropriately used visual directions as well as simple and clear linguistic directions, so the participants could practice exercise motions with desirable posture and finally learn successful exercising techniques^35,36^. Positive improvement in lower- and upper-limb muscle endurance and aerobic endurance capacity deems to appear due to those elemental factors.

The American college of Sports Medicine and the American Heart Association recommend, in ‘Recommendations for physical activities in elderlies’, aerobic exercise with moderate intensity (30 min., 5 times/week) and high intensity (20 min., 3 times/week) and muscle strengthening exercise with 8-10 motions (10-15 repetition/motion, 2-3 times/week), as an exercise prescription for >65 yearold elderlies to improve muscle strength as well as endurance^37^. The SBHE program conducted in this study at this point of view is considered to comply with appropriate intensity of exercise that improvement of muscle endurance is expected. When looking at other relevant preceding studies, Kim^38^ reports that motion indexes such as sitting-on and standing-up from the chair, going-into and -out from the toilet, bathtub and shower stall, walking 50 m on the even ground, etc. were improved by the 3 times a week, 24-week physical stimulating exercise program for elderlies with dementia. In addition, it was shown to match to the study results that the 12-week yoga program improved muscle strength, muscle endurance and body endurance in elderlies with dementia^39^ and the complex exercise combining aerobic and elastic band exercises increased physical fitness factor in elderlies with dementia^40^. For flexibility, it is recommended to do flexibility exercise more than twice a week^41^. As same to the other physical fitness factors, flexibility decreases with aging^42^, while the risk of falling increases with reduced joint working range as well as decreased physical activities^43^. For stretching as a way to improve flexibility, positive results can be obtained from intermittent stretching performed during exercise rather than that performed before exercise. As static stretching decreases efficacy of exercise as muscle- weakening continues for 15 minutes loosening sinews and muscles, stretching at the warm-down stage when the temperature of muscles increases after main exercise is recommended^41,44^.

In this study, dynamic stretching was performed following the rhythm of music at the warm-up stage and static stretching was performed at the warm-down stage, resulting in no positive change in terms of flexibility. Such result might be because stretching was performed to recover body condition at the warm-down stage while static stretching to increase ROM by gradually and smoothly relaxing muscles and connective tissues was not continued for sufficient time. Therefore, positive change in terms of flexibility can be expected if intentional and phased static stretching program to stimulate unique attributes of muscles and joints movable to the maximum range is included in the warm-down stage or extra stretching program is added.

Secondly, the SBHE program caused increase in MMSE-K point and BDNF concentration in the exercise group, compared to the control group. It might be difficult to assess the normal value of BDNF concentration as the absolute value has not been not reported while MMSE-K point indicated normal level of cognitive capacity in both group (control group=25.19±1.83; exercise group=26.23±1.64); after applying the SBHE program, cognitive capacity was found to statistically significantly increase in the exercise group (27.08±2.10), which confirms improvement of cognitive capacity. According to preceding studies regarding effect of exercise on cognitive function, the complex exercise including 16-week aerobic, muscle strength, balance and flexibility exercises increased cognitive function in >65 year-old elderlies while 12-week aerobic and elastic band exercise increased serum BDNF concentration in female elderlies^16^, which matches to the results of this study. Despite difficulty in direct comparison between preceding studies and this study, Han *et al*^28^ reports that 12-week aerobic exercise was conducted in >65 year-old elderlies, and as a result, cognitive capacity decreased after the exercise compared to that before exercise in the control group (-1.2±1.5), indicating improvement of cognitive capacity with increase by 0.6±1.6 27 from 26.6±2.1 before the exercise, to 27.2±2.0 after the exercise. Han *et al*^28^ also choose the elderlies with normal cognitive function as the study subjects and the resulting MMSE-K point was very similar to the results of this study, reporting improvement of cognitive capacity by 12-week aerobic exercise. In short, the SBHE program consisted of exercises with moderate-high intensity which increase easy supply of oxygen and nutrients as well as removal rate of waste matter and carbon dioxide, resulting in decrease in depression, anxiety and nervous tension as well as improvement of cognitive capacity^45^. In addition, the SBHE program has positive effect on cognitive capacity, as it proceeds with repeated simple motions and gradually progresses complex motions and include learning activities through communication with the instructor.

Hyodo *et al*^46^ reports that cognitive function through lateralized frontal activation has correlation to high ability to aerobic exercise, suggesting that improvement of ability to aerobic exercise is closely related to improvement of cognitive capacity. Consequently, it supports the result of study done by Kemoun *et al*^47^ that long-term based aerobic exercise improves cognitive capacity. As mentioned above, even though there are a number of studies reporting improved cognitive capacity accompanied by improved aerobic exercise ability, physiological mechanism regarding correlation between aerobic exercise ability and cognitive capacity has not been clearly known so far. However, regular exercise has been reported to promote growth and survival of brain nerve cells by improving cerebral blood flow while decreasing brain tissue atrophy, resulting in improvement of cognitive capacity^48,49^. It suggests various growth factors (BDNF, IGF-1, VEGF, NGF) might play important role^50,51^. Expression of BDNF increases via exercise^49,52^, which contributes to growth, differentiation and survival of nerve cells, consequently improving cognitive capacity^53^. The results of this study shows no relationship between cardiopulmonary endurance and change in cognitive capacity (no data presented). Undoubtedly, precaution needs to be taken in interpretation of correlation between aerobic exercise ability and cognitive capacity, as the number of subjects in this study was small; further study based on large-scale relevant cases seems to be required.

The SBHE program of this study was conducted in the way to learn how to memorize motions during the process of motion combination by step. This learning method is believed to cause positive change in cognitive function in elderlies by inducing cognitive response to memory recall, verbal language and sign language instructions. In addition, it is determined to improve physical fitness factors of lower- and upper-limb muscle endurance and aerobic endurance by repeating motions and gradually increasing the intensity of exercise depending on the pattern of motions. However, no improvement of flexibility, dynamic balance and agility appeared, suggesting need to add extra program to improve these factors. In addition, music is known to improve coordination of exercise by decreasing fatigue, increasing the level of psychological awakening and limiting physically uncomfortable senses^54^, consequently having consistent effect on cardiopulmonary response^55^. Especially by helping to do exercise with appropriate speed and intensity, music can positively influence overall exercise experiences^25^. The exercise program using music has been reported from various studies to have an effect on improvement of physical strength and cognitive function in elderlies^56-58^. Undoubtedly, as the effect of music cannot be directly discussed in this study, further relevant study might be needed.

## CONCLUSION

This study was conducted to find out the effect of exercise on physical fitness, cognitive function and brain-derived neurotrophic factors (BDNFs) by applying the 12- week SBHE program to the 65-79 year-old elderlies, in order to verify feasibility of exercise program for largescale geriatric study. Accordingly, the results of this study are as the following. The 12-week SBHE program improved lower- and upper-limb muscle endurance as well as aerobic endurance, except flexibility, dynamic balance and agility, in the exercise group, compared to the control group. In addition, improvement of cognitive function was confirmed by MMSE-K test and BDNF concentration was shown to increase in the exercise group. As the results mentioned above, the SBHE program was determined to consist of appropriate exercise motions with appropriate intensity required for elderlies and is considered to be suitable program for large-scale study to verify change in physical fitness, cognitive function and brain derived neurotrophic factors of elderlies if modified and supplemented to add motions for flexibility, dynamic balance and agility. In addition, improvement of cognitive function is expected to be clearly confirmed if applying this exercise program to subjects “dementia suspected” (MMSE-K point: 20-23) rather than to subjects with normal cognitive function.
